# Quantifying Reoxygenation in Pancreatic Cancer During Stereotactic Body Radiotherapy

**DOI:** 10.1038/s41598-019-57364-0

**Published:** 2020-01-31

**Authors:** Edward Taylor, Jitao Zhou, Patricia Lindsay, Warren Foltz, May Cheung, Iram Siddiqui, Ali Hosni, Ahmed El Amir, John Kim, Richard P. Hill, David A. Jaffray, David W. Hedley

**Affiliations:** 1 Radiation Medicine Program, Princess Margaret Cancer Centre, 610 University Avenue, Toronto, Ontario M5G 2M9 Canada; 2 Department of Abdominal Oncology, Cancer Center and Laboratory of Signal Transduction and Molecular Targeting Therapy, West China Hospital, Sichuan University, Chengdu, China; 3 Ontario Cancer Institute, Princess Margaret Cancer Centre, 610 University Avenue, Toronto, Ontario M5G 2M9 Canada; 4 Department of Pathology, Hospital for Sick Children, 555 University Avenue, Toronto, Ontario M5G 1X8 Canada; 5 Division of Medical Oncology and Hematology, Princess Margaret Cancer Centre, 610 University Avenue, Toronto, Ontario M5G 2M9 Canada

**Keywords:** Biophysics, Cancer, Oncology

## Abstract

Hypoxia, the state of low oxygenation that often arises in solid tumours due to their high metabolism and irregular vasculature, is a major contributor to the resistance of tumours to radiation therapy (RT) and other treatments. Conventional RT extends treatment over several weeks or more, and nominally allows time for oxygen levels to increase (“reoxygenation”) as cancer cells are killed by RT, mitigating the impact of hypoxia. Recent advances in RT have led to an increase in the use stereotactic body radiotherapy (SBRT), which delivers high doses in five or fewer fractions. For cancers such as pancreatic adenocarcinoma for which hypoxia varies significantly between patients, SBRT might not be optimal, depending on the extent to which reoxygenation occurs during its short duration. We used fluoro-5-deoxy-α-D-arabinofuranosyl)-2-nitroimidazole positron-emission tomography (FAZA-PET) imaging to quantify hypoxia before and after 5-fraction SBRT delivered to patient-derived pancreatic cancer xenografts orthotopically implanted in mice. An imaging technique using only the pre-treatment FAZA-PET scan and repeat dynamic contrast-enhanced magnetic resonance imaging (DCE-MRI) scans throughout treatment was able to predict the change in hypoxia. Our results support the further testing of this technique for imaging of reoxygenation in the clinic.

## Introduction

Surgical resection is possible in 15–20% of patients with a new diagnosis of pancreatic cancer and provides the only chance of long-term cure, even though local recurrence or distant metastases occur in the majority of surgically treated patients. Although pancreatic cancers can be highly metastatic, in a proportion of patients, clinical outcome is dominated by local disease progression. Conventional external beam radiotherapy, alone or in combination with chemotherapy, can provide short term local disease control and relief of symptoms, but is not curative. Stereotactic Body Radiotherapy (SBRT) delivers a small number of conformal, high-dose fractions to the tumour. It has the potential to improve patient quality of life through better local control (due to higher ablative doses being delivered) and shorter course of treatment, and is of current clinical interest^1–5^. However, similar to conventional radiotherapy, SBRT is limited by the radiation sensitivity of adjacent normal tissues, and the relative radio-resistance of pancreatic cancer.

Hypoxia is widely believed to be a major component of radio-resistance in solid tumours^6^. Conventional fractionation that extends treatment over several weeks in principle allows time for reoxygenation^7^. The extent to which reoxygenation occurs during the relatively short duration of SBRT is unclear. Our recent work using the nitroimidazole tracer pimonidazole to measure the extent of hypoxia in surgical specimens of pancreatic cancer by quantitative immunohistochemistry and FAZA-PET^8^, highlights the substantial degree of variation in hypoxia between individual pancreatic cancers. We hypothesize that the extent of hypoxia and degree of reoxygenation during SBRT will be important determinants of treatment efficacy^9^, as they are for conventional fractionated radiotherapy^10,11^.

These two factors—the significant inter-patient variability of hypoxia and its impact on radiotherapy efficacy—provide a rationale for personalizing treatment, optimizing the dose fractionation schedule to accommodate temporal heterogeneities in tumour hypoxia and incorporating targeted agents such as hypoxia activated pro-drugs (HAPs) to target residual radio-resistant subpopulations^12,13^. HAPs are a class of compounds that are metabolically activated to cytotoxic agents under low oxygen conditions, and hence, primarily target a different subpopulation than SBRT^14^, which is most effective at killing well-oxygenated cells^15^. Patient-specific optimization of HAP and SBRT dose fractionation schedules to maximize cell-killing in hypoxic tumours thus requires: 1.) radiobiological modeling of treatment response that incorporates reoxygenation and proliferation kinetics^16–18^ and 2.), an imaging measurement and analysis framework to characterize treatment response *in vivo*, including oxygenation status, which can readily be incorporated into a routine clinical workflow.

These requirements provided the impetus for us to study the reoxygenation of pancreatic tumours during SBRT, and to develop an imaging technique to measure reoxygenation. An additional goal of this work was to develop a pre-clinical model of pancreatic cancer that could be used to study the interactions between HAPs, SBRT, treatment delivery scheduling, and reoxygenation kinetics. Orthotopically-grown patient-derived xenografts were treated with SBRT (35–45 Gy in 5 fractions, delivered every other day), delivered precisely under image guidance using a micro-irradiator, simulating the protocol being used in our cancer centre to treat pancreatic cancer patients. Reoxygenation was assessed using repeat FAZA-PET scans (before and after SBRT) and DCE-MRI to study the relationship between reoxygenation and perfusion changes. Quantification of reoxygenation using DCE-MRI acquired during treatment would be easier to implement clinically than repeat FAZA-PET imaging and hence, may enable the large-scale acquisition of data on which to base treatment optimization models. There is an emerging capacity at our cancer centre to image patients on a hybrid PET-MRI scanner and to deliver pancreatic SBRT safely under MR-guidance^19^ and so the primary goal of this work was to demonstrate the feasibility of measuring reoxygenation during SBRT and to employ DCE-MRI to quantify it in a pre-clinical setting. The results provide support for further refinement of the imaging technique and modelling of reoxygenation at the clinical scale.

## Results

### Response of OCIP23 and SGPC35 models to SBRT

**Figure 1:**
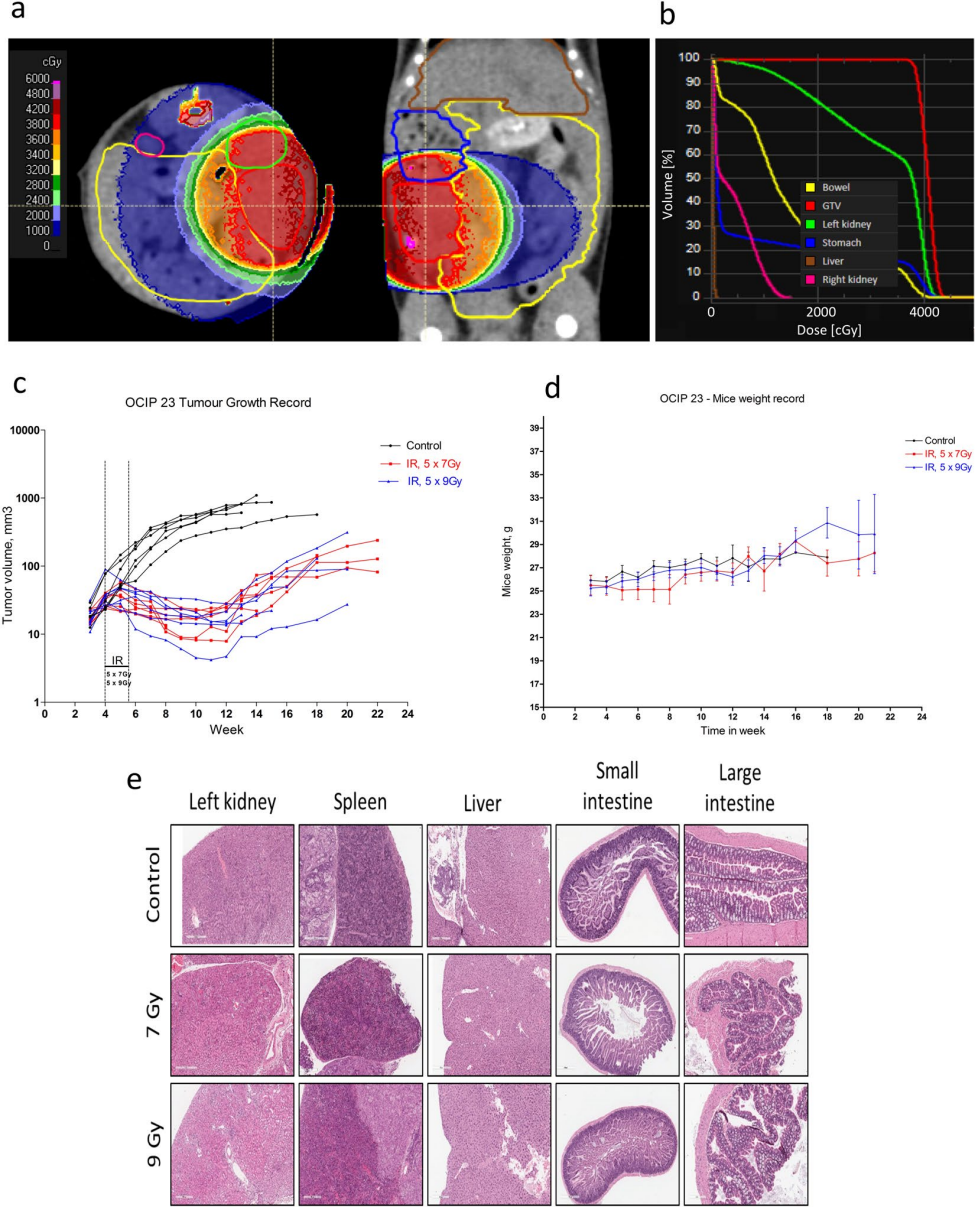
(**a)** Dose distribution for a representative mouse plan (40 Gy prescription; 225 kVp photons;15 mm collimator) using a 180 degree arc delivery (see text for details). Tumour gross tumour volume (GTV; red), bowel (yellow), left kidney (green), right kidney (fuchsia), liver (brown), and stomach (blue) are delineated by lines of indicated colours. Dose is overlaid as a color wash. **(b)** Dose-volume histogram for the tumour GTV and organs-at-risk. **(c)** Volumes of orthotopically-grown OCIP23 xenografts measured by MRI. **(d)** Animal weights following treatment. **(e)** Representative H&E sections from adjacent normal tissues from non-irradiated control mice and mice receiving 7 and 9 Gy.

The initial work used the OCIP23 patient-derived xenograft (PDX), which has typical features of pancreatic cancer and has been extensively studied in the laboratory^13,20–22^. The dose distribution from a representative treatment plan is shown in Fig. 1a,b. As seen in Fig. 1c, there was a reduction in tumour volume following 5 fractions of 7 Gy or 9 Gy delivered every second day, with significant growth delay compared to the non-irradiated controls. The treated mice gained weight at a similar rate to the controls (Fig. 1d), and no evidence for radiation damage was observed in histological sections obtained from adjacent normal tissues (Fig. 1e), even though substantial fractions of bowel, stomach and left kidney received the full prescription dose (Fig. 1b). However, during post-treatment follow up, many of the OCIP23 mice developed ulcerated cutaneous nodules near the incision used for the orthotopic implants, rendering this model unsuitable for studies required prolonged monitoring.

**Figure 2:**
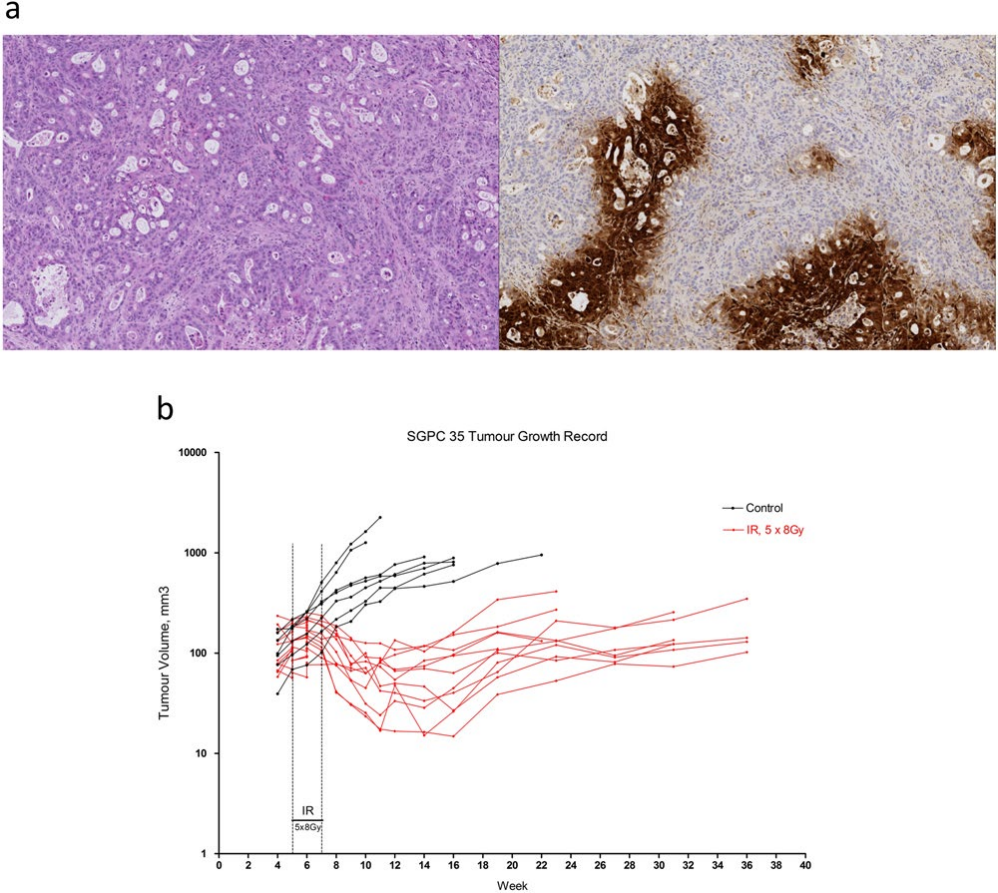
(**a)** Histological features of SGPC35 patient-derived xenografts grown orthotopically stained with H&E (top left), and by immunohistochemistry to reveal the hypoxia tracer pimonidazole (top right). **(b)** Tumour volumes of irradiated and control SGPD35 xenografts, measured by MRI, over time.

The SGPC35 PDX model is hypoxic (Fig. 2a) and grows readily at the orthotopic site. Treatment with 40 Gy delivered in 5 fractions every second day led to a pronounced suppression in tumour growth relative to the control arm (Fig. 2b). Mice were sacrificed once their tumours reached ≈750 mm^3^ or were deemed to be in distress. Apart from a single mouse that developed a fast-growing ulcerated cutaneous nodule near the incision (and likely outside the irradiated volume), the irradiated mice survived until a time between 119 and 219 days after radiotherapy. In contrast, most of the control mice were sacrificed between 50 and 100 days after reaching treatment size, with only one surviving to 127 days.

### Hypoxic fraction was reduced by irradiation

**Figure 3:**
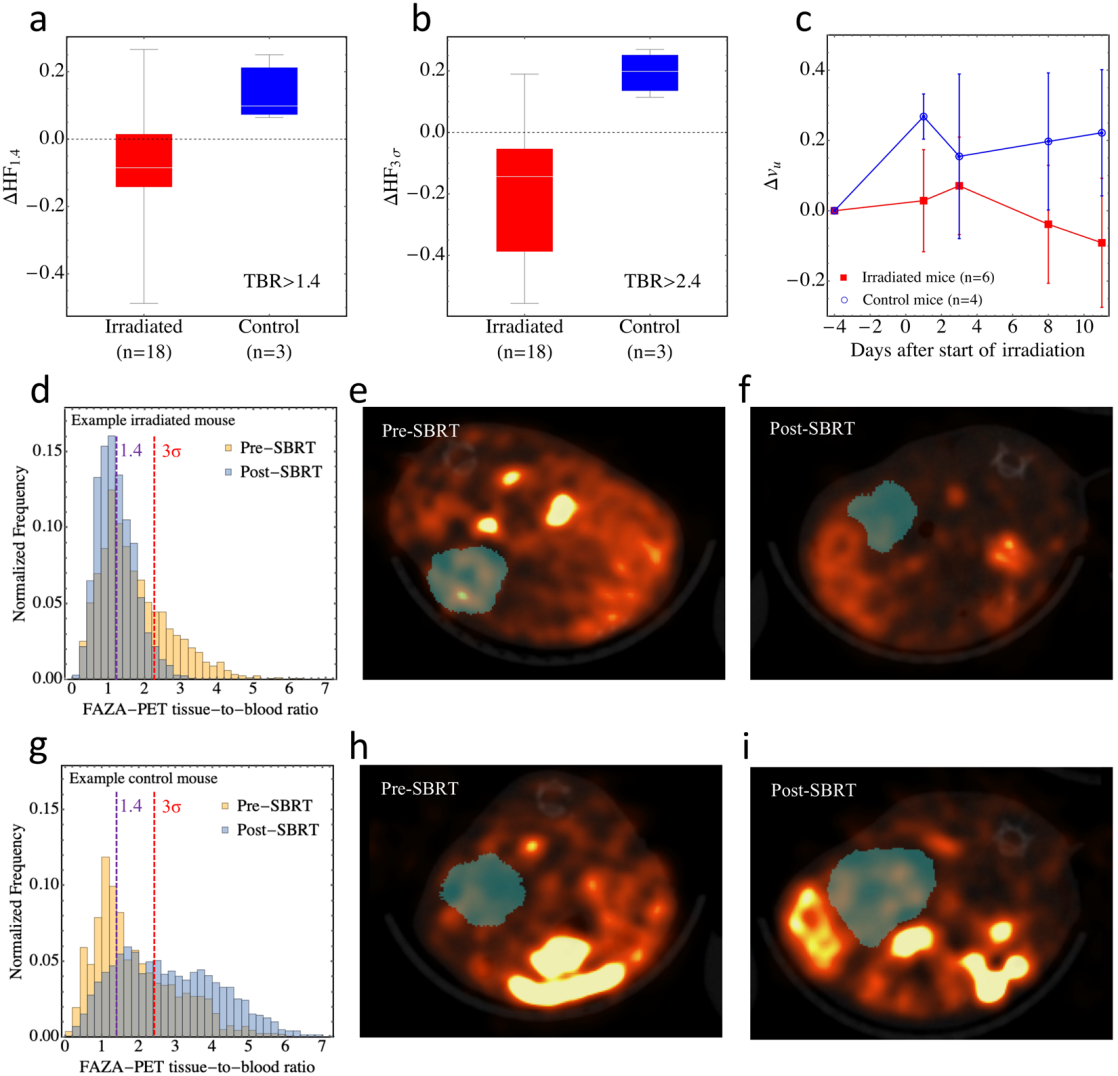
Box and whisker plots showing the change in hypoxic fractions using a threshold TBR > 1.4 **(a)** and TBR > 2.4 **(b)** over the course of SBRT for the irradiated (n = 18) and control (n = 3) mice from both SGPC35 cohorts that were subject to FAZA-PET imaging. Radiation reduced hypoxia in the irradiated mice, while hypoxic fraction increased over the same time period in the control mice. **(c)** Change in the volume fraction *v*_*u*_ of unperfused tissue over the course of radiotherapy for irradiated (n = 6) and control mice (n = 4) from the second cohort that were imaged during treatment with DCE-MRI. (Note that of the 4 control mice that were imaged, all 4 were subject to DCE-MRI but only 3 underwent FAZA-PET imaging). Error bars indicate standard deviations at each time points across the 6 irradiated and 4 control mice. *v*_u_ decreased for the irradiated mice and increased for the control mice. **(d)** Voxel-scale histogram of the FAZA tissue-to-blood ratio (TBR) before and after SBRT for a representative irradiated tumour; vertical dashed lines indicate TBR values of 1.4 and 3σ (=2.42) used for hypoxic fraction quantification. FAZA uptake in an axial slice through the same tumour (blue shaded region) before **(e)** and after **(f)** SBRT. **(g–i)** As above for a representative non-irradiated control tumour.

Mean hypoxic fractions (based on FAZA-PET imaging) of all irradiated SGPC35-implanted mice (n = 18, including mice from two SGPC35 cohorts, one with repeat DCE-MRI during treatment and one without; but both with FAZA-PET imaging see Methods for experimental details.) before irradiation were HF_3σ_ = 0.33 (population standard deviation σ = 0.17) and HF_1.4_ = 0.66 (σ = 0.13), where ${{\rm{HF}}}_{\Lambda }$ indicates the fraction of PET imaging voxels for which the mean activity divided by that in blood—the tumour-to-blood ratio (TBR)—exceeds the threshold $\Lambda$. (3σ = 2.42 is derived from muscle statistics; see Methods.) Twenty-four hours after completion of SBRT, the hypoxic fractions were reduced: HF_3σ_ = 0.15 (σ = 0.15) and HF_1.4_ = 0.58 (σ = 0.16). For the control mice that were imaged using FAZA-PET (n* = *3), hypoxic fractions increased over the same time span: HF_3σ_ = 0.25 (σ = 0.04) to HF_3σ_ = 0.45 (σ = 0.04) and HF_1.4_ = 0.59 (σ = 0.05) to HF_1.4_ = 0.73 (σ = 0.09); see Fig. 3a,b. Amongst irradiated tumours, mean tumour-scale TBR was reduced from 2.06 (σ = 0.44) to 1.69 (σ = 0.37). For control tumours, TBR increased from 1.83 (σ = 0.08) to 2.42 (σ = 0.35). Figure 3d shows the voxel-scale histogram of the FAZA tissue-to-blood ratio (TBR) before and after SBRT for a representative irradiated tumour (vertical dashed lines indicate TBR values of 1.4 and 3σ (=2.42) used for hypoxic fraction quantification). This figure exhibits a general trend observed for the majority of irradiated tumours: the primary impact of radiotherapy is to remove the high-FAZA uptake “tail” (TBR ≳ 3) of the histogram, corresponding to reoxygenation of the most hypoxic tissue voxels. Figure 3e,f shows FAZA uptake in an axial slice through the same tumour (blue shaded region) before (e) and after (f) SBRT, and Fig. 3g–i shows representative results from a non-irradiated control tumour (“pre-SBRT” and “post-SBRT” in this panel refer only to the time points).

**Figure 4:**
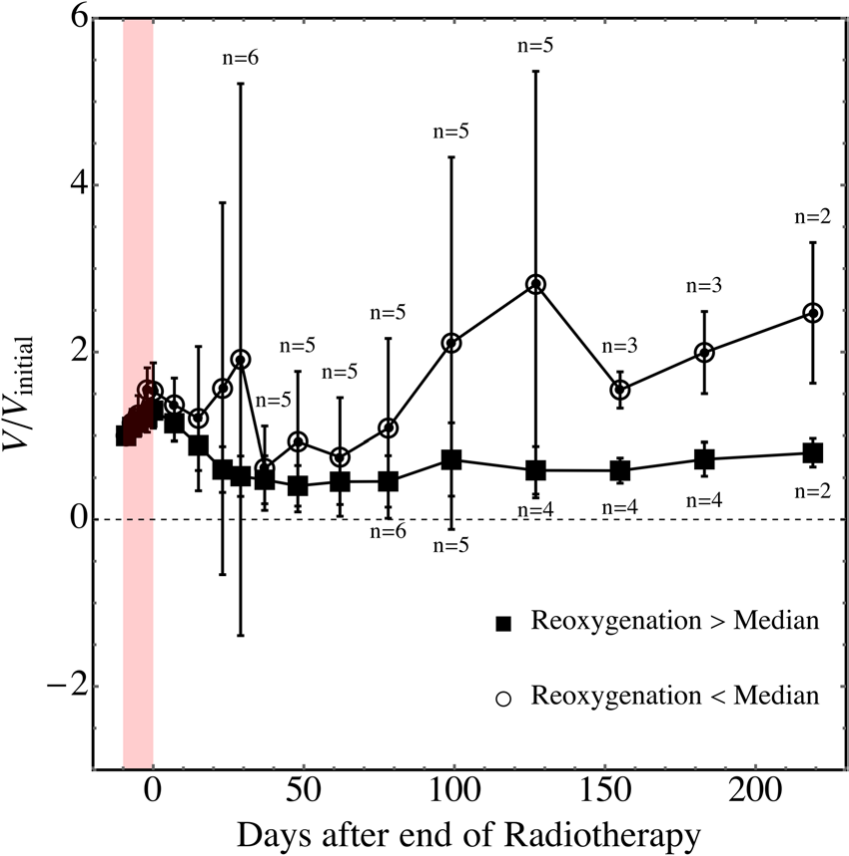
Tumour growth inhibition was greater for tumours that reoxygenated more during radiotherapy. Mean scaled tumour volumes—volumes divided by volumes at the start of radiation—are shown as a function of time after the end of radiotherapy for irradiated tumours segregated into two groups based on reoxygenation: ΔHF_1.4_ < −0.09 (the median value for ΔHF_1.4_) and ΔHF_1.4_ > −0.09. Error bars indicate the standard deviation of scaled tumour volumes in each group. The number of mice n in each group are shown above and below error bars; where not shown, n = 6. The analogous plots using ΔHF_3*σ*_ are essentially the same. Mann-Whitney *U* test *p*-values were 0.08, 0.12, 0.02, and 0.02, 0.053 at 50, 100, 127, 183, and 219 days after completion of SBRT, respectively. The test was not significant (*p* > 0.05) at the last volume measurement (219 days post-SBRT) because only 4 mice lived to this time.

Amongst irradiated SGPC35-implanted mice from the first cohort (n = 12; no DCE-MRI during treatment), which were followed after SBRT to assess growth inhibition (Fig. 2b), tumour growth inhibition was more pronounced for tumours that reoxygenated more during SBRT (Fig. 4).

### PET-MRI imaging of reoxygenation

An MRI-based reoxygenation imaging technique was developed using voxel-scale MRI dynamic contrast enhancement patterns as a surrogate for SBRT-induced perfusion changes, based on fitting of the per voxel signal enhancement to a bimodal distribution of perfused and unperfused states (Eq. 1, Methods). Changes in the unperfused volume fraction, ${v}_{u}$, were then combined with pre-treatment FAZA-PET measurements of hypoxic fraction for 10 mice (6 irradiated mice, 4 control, comprising the second SGPC35 cohort; see Methods for details) to predict the change ${\Delta {\rm{HF}}}_{\Lambda ,{\rm{predicted}}}$ in hypoxic fraction, as per Eqs. 2 and 3 (Methods).

**Figure 5:**
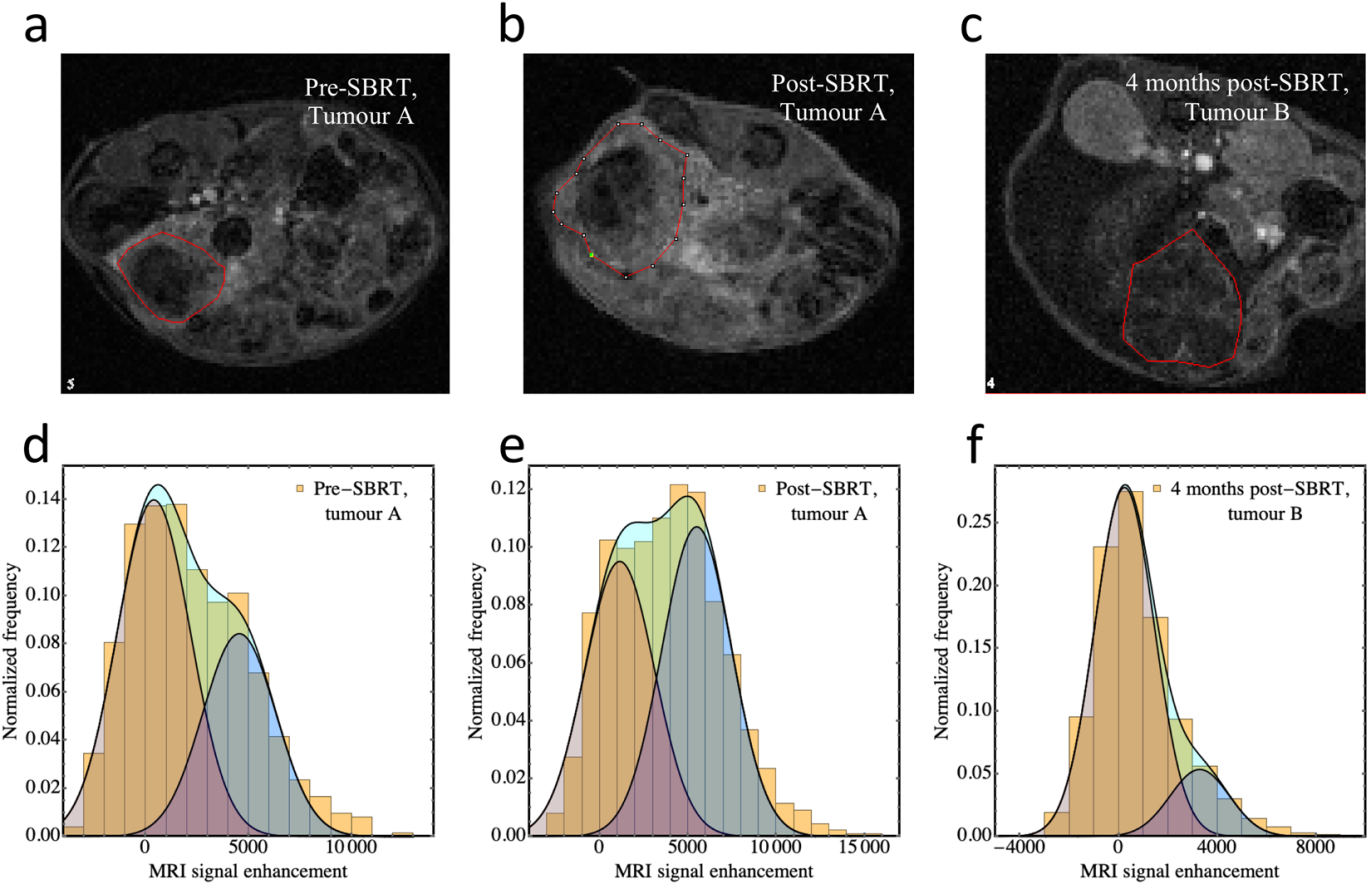
Bimodal MR signal enhancement in SPGC35 mice. Representative DCE-MR images (1 minute post-injection) before **(a)**, after **(b)** SBRT from a second-cohort mouse, and from a first-cohort mouse at 4 months post-SBRT **(c)**. The tumour contours are shown by the red lines. The spatial enhancement patterns are suggestive of high levels of solid stress in central regions, occluding blood vessels pre-SBRT, partial central de-occlusion post-SBRT, and substantial hypoperfusion throughout the entire tumor late post-SBRT. **(d–f)** Example bimodal fits (solid lines) to binned voxel-scale MRI signal enhancement data 60 s after contrast injection for the full volumes of the tumours shown in **(a–c)**. The unperfused volume fractions *v*_*u*_ from the fits are: 0.44, 0.17, and 0.63 respectively. The individual “modes” in the bimodal fits—as well as their sums—are presented as separate filled distributions.

Representative images of the spatially heterogenous contrast enhancement patterns are presented in Fig. 5. All tumours exhibited substantial enhancement in the peripheral region of the tumour and hypo-enhancing cores prior to SBRT, indicating a central paucity of patent, functional blood vessels (Fig. 5a) SBRT increased enhancement in the tumour cores (Fig. 5b), whereas in control tumours, the cores became increasingly hypo-perfused over the same time period. Late post-SBRT, tumors presented with more uniform hypoperfusion (Fig. 5c).

**Figure 6:**
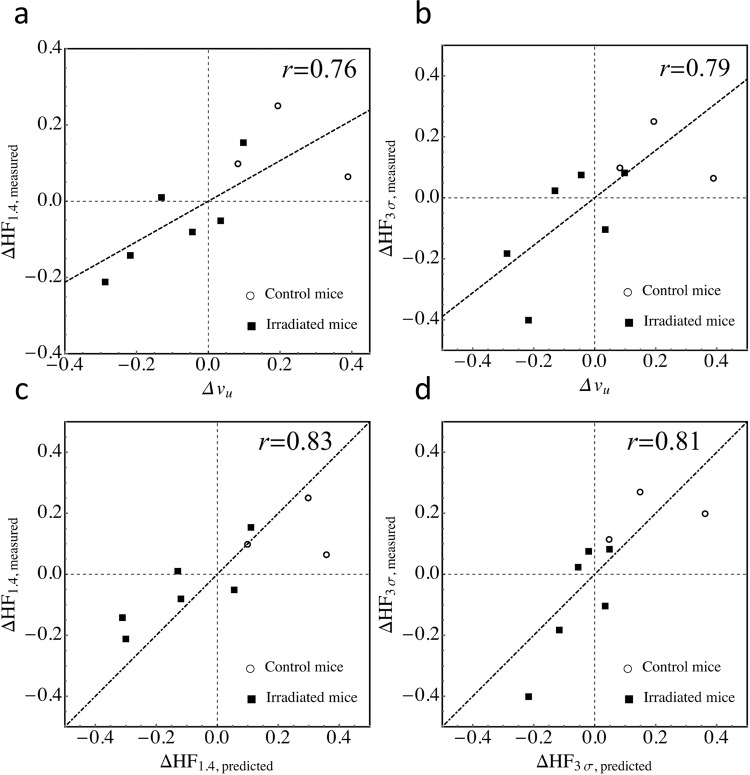
Measured changes in hypoxic fraction (post SBRT minus pre SBRT) versus changes in volume fraction ${v}_{u}$ of unperfused tissue using two thresholds, TBR > 1.4 **(a)** and TBR > 2.4 (**b)** used to determine PET hypoxic fraction for the 9 mice (6 irradiated, 3 control) that underwent both DCE-MR and FAZA-PET imaging. ${v}_{u}$ decreases during SBRT in irradiated tumours, indicating a drop in perfusion heterogeneity. In turn, hypoxic fraction is reduced by the increased oxygenation. **(c,d)** Measured changes in hypoxic fraction versus the values predicted from baseline HF values and pre- and post-SBRT ${v}_{u}$ values for the thresholds. The dashed lines in the bottom plots are equal to unity (i.e., lines of perfect prediction) and are not fits. The fact that the apparent “slope” of the measured vs predicted HF data for the threshold of 1.4 is less than one suggests that for this relatively low threshold, a nonzero value of ${{\rm{HF}}}_{0}$ (see Methods) would be a better approximation. Conversely, for the predicted HF values corresponding to the higher threshold, the assumption of ${{\rm{HF}}}_{0}\approx 0$ is evidently good (even though correlations are incrementally smaller).

Consistent with these qualitative observations, ${v}_{u}$ was found to decrease during SBRT from 0.47 (σ = 0.12) to 0.38 (σ = 0.14) for the n = 6 irradiated mice. In n = 4 control tumours over the same time, ${v}_{u}$ increased from 0.35 (σ = 0.04) to 0.57 (σ = 0.12). (Figs. 3c, 5d,e). The corresponding changes in measured hypoxic fraction between serial PET-FAZA scans (${\Delta {\rm{HF}}}_{\Lambda ,{\rm{measured}}}$) correlated with changes in ${v}_{u}$ (Pearson’s *r* = 0.76 and 0.79 for $\Lambda =1.4\,{\rm{and}}\,3{\rm{\sigma }},\,{\rm{respectively}}$; Fig. 6a,b). The ${\Delta {\rm{HF}}}_{\Lambda ,{\rm{measured}}}$ were strongly correlated with the predicted values $\,{\Delta {\rm{HF}}}_{\Lambda ,{\rm{predicted}}}$ (Pearson’s *r* = 0.83 and 0.81 for $\Lambda =1.4\,{\rm{and}}\,3{\rm{\sigma }},\,{\rm{respectively}}$; Fig. 6c,d).

## Discussion

Evidence is emerging that dose escalation (delivering $\gtrsim$70 Gy biologically effective dose) for patients with locally advanced pancreatic cancer can improve overall survival^1,4,23^. This includes the 40 and 45 Gy in 5 fractions dose schedules used in the present work, for which the biologically effective doses (BED) were 72 and 85.5 Gy, respectively, assuming *α*/*β* = 10 Gy. At the same time, hypoxia varies greatly amongst pancreatic cancer patients^8^, meaning that, accounting for the enhanced radio-resistance of hypoxia cells via the oxygen enhancement ratio (OER)—the true effective dose may in some cases be much less than expected, conferring a survival advantage to hypoxic—and possibly more malignant^20,24–27^—cells. In order to accurately model the response of pancreatic tumours to hypofractionated radiotherapy and hypoxia-activated prodrugs^14,16,17,28^, and to develop strategies to improve survival, it will be important to accurately quantify hypoxia and reoxygenation during the short course of SBRT^7^.

In this work, a patient-derived xenograft pancreatic cancer model (SGPC35) was used to study reoxygenation during SBRT and to develop an image measurement and analysis framework to quantify reoxygenation which can readily be incorporated into routine clinical workflow. Using FAZA-PET hypoxia imaging, hypoxic fractions were found to decrease in irradiated tumours versus non-irradiated ones (Fig. 3), indicating that reoxygenation occurred even during short-duration SBRT, albeit using a schedule where radiation was delivered every other day. Every-other-day delivery of SBRT is sometimes implemented clinically to reduce normal tissue toxicities; here, it likely allowed greater time for reoxygenation as well. Although change in hypoxic fraction using the lower, more standard metric of tumour-to-blood FAZA activity ratio $>$1.4 was small, the change in hypoxic fraction using a ratio of 2.4 (three times the standard deviation of pooled muscle activity values) was much larger. This means that reoxygenation was greatest amongst the most hypoxic voxels (see e.g., Fig. 3d). Supporting the idea that the OER effect may be important in determining the response of pancreatic tumours to radiotherapy, growth inhibition was greatest for tumours exhibiting high levels of reoxygenation during radiotherapy (Fig. 4).

The differences in hypoxic fraction before and after SBRT were well correlated with the changes in the volume fraction ${v}_{u}$ of tissue that was poorly perfused, as determined using DCE-MRI (Fig. 6a,b). This is noteworthy since the decrease in ${v}_{u}$ that accompanied reoxygenation in irradiated tumours described an *increase* in the perfusion of the central, hypo-enhancing tumour cores, relative to the well-perfused periphery; i.e., spatial heterogeneity of perfusion decreased during SBRT. In contrast, radiation-induced inflammation^29,30^, which has been proposed as a mechanism for acute reoxygenation resulting from vasodilation, would result in a more uniform perfusion enhancement or, if the central tumour regions are necrotic, increased perfusion enhancement in the tumour periphery^31^.

A possible explanation for the observed pattern of perfusion enhancement is that blood vessels were occluded in the tumour core as a result of solid stress^32–34^ or other related forms of pressure^35–37^. Indeed, it has been estimated that ≈75% of blood vessels in pancreatic tumours are occluded^38,39^, likely contributing to hypoxia. Solid stress in pancreatic tumours arises from the presence of a stiff gel comprised of hyaluronic acid and collagen in the tumour stroma^32^. Strain induced by proliferating epithelial and stromal cells is transmitted by this gel through the stroma, occluding the blood vessels there once a critical value of stress is exceeded^33^. Tumour cell depletion in pre-clinical models using diphtheria toxin has been shown to reduce solid stress and de-occlude blood vessels^32^. It stands to reason that radiation-induced cell death involving loss of cell volume (i.e., excluding senescence) would also decrease blood vessel occlusion, leading to an increase in oxygenation. Solid stress is greatest in the tumour centre and occlusion occurs primarily there^32,33,40^. Thus, a reduction in solid stress due to radiation-induced cell death would lead to diminished blood vessel occlusion primarily in the central regions of tumours, consistent with a decrease in ${v}_{u}$.

Irrespective of the mechanism underlying perfusion enhancement, our model [Eqs. (1–3)] was able to predict changes in hypoxic fraction from the DCE-MRI scan, using data from near-concurrent FAZA-PET and MRI scans acquired before SBRT began (Fig. 6c,d). This suggests that reoxygenation could be quantified in the clinic without the need for serial PET imaging. Given that MRI is increasingly being incorporated into the standard image-guided radiotherapy workflow^41^, including the use of combined MR scanners and linear accelerators (or “MR-linacs”), this would make it easier to acquire data that could provide radiobiologically-based tumour response models as well as eventually allow for the adaptation of treatment during the course of radiotherapy to mitigate OER effects.

DCE-MRI quantifies properties of the vasculature^42^ and studies have demonstrated correlations between quantitative pharmacokinetic DCE-MRI parameters such as *K*^trans^ and hypoxia in clinical studies of cervical^43,44^, head and neck^45^, and pre-clinical pancreatic^46^ cancers. A shortcoming of DCE-MRI is that it only accounts for capacity to deliver oxygen and not tumour metabolism, potentially diminishing correlations between it and hypoxia^47,48^. Hence, the need to “calibrate” DCE-MRI to FAZA-PET in our model, to account for differences in oxygen metabolism. Additional shortcomings of DCE-MRI include the complexity of pharmacokinetic data analysis and hence, sensitivity to differences in data collection (e.g. biases from radiofrequency non-uniformity, arterial input function, and endogenous T1 mapping measurements)^49–52^ and post-processing^53^.

Other MRI-based schemes to quantify hypoxia include diffusion-weighted MRI (DWI) scans performed over different ranges of magnetic field gradient values to assess perfusion (inter-voxel incoherent motion) and metabolism (apparent diffusion coefficient) separately^48^, repeat MR scans which quantify spin relaxation changes under different oxygenation conditions^54–56^, quantitative BOLD imaging^57,58^, and pattern recognition analysis of DCE-MRI^59–61^. By-products of metabolism have also been imaged using ^13^C-hyperpolarized MRI^62^ and magnetic resonance spectroscopy^63,64^. Reflecting the different biology of different tumour types, it is likely that there is no “one size fits all” solution for MRI-based hypoxia imaging (apparent diffusion for pancreatic tumours is sensitive to stromal density/cellularity^65^—and hence, metabolism—but also fibrosis^66^, for instance), and imaging schemes that are easily incorporated into clinical workflows will likewise vary from site to site. Disease site-specific MRI-based biomarkers will need to be developed for different sites.

The imaging technique developed in this paper satisfies the requirement of easy incorporation into the clinic, potentially facilitating the large-scale acquisition of reoxygenation data for pancreatic cancer patients receiving radiotherapy. Although our imaging study involved a single mouse model and a small number of mice, we believe that it will be more relevant to fine-tune the technique described here, including the potential use of inter-voxel incoherent motion (IVIM) MR imaging to assess perfusion and oxygen metabolism changes^48^, in the clinic rather than continuing to develop it pre-clinically, since MR imaging of small animals presents unique challenges^67^ that are distinct from those found in clinical imaging. At the same time, our centre has the capacity to acquire functional PET-MRI imaging (IVIM, DWI, contrast-MRI, FAZA-PET) before and after treatment, as well as on-board functional imaging—including DWI and contrast MRI—of patients on an integrated MR-linac. With the goal of building on-board functional imaging capacity to develop treatment response models, we are currently setting up a clinical trial to apply the imaging and treatment workflow described here to pancreatic cancer patients undergoing SBRT on an MR-linac.

## Methods

### Overview of experimental design

All methods were performed in accordance with the relevant guidelines and regulations of the University Health Network. Patient-derived xenografts designated OCIP23 and SGPC35 were established from pancreatectomy samples superfluous to patient diagnosis as previously described, using a protocol approved by the University Health Network Research Ethics Board^20^. Informed consent was obtained from participating patients. The patient-derived xenografts were maintained by serial *in vivo* subcutaneous passage and implanted at the orthotopic site by suturing small cubes of tissue on the surface of the exposed pancreata of NRG mice according to protocols approved by the University Health Network Animal Care Committee. To study the effects of SBRT, OCIP23-implanted mice were treated with 5 fractions on a Mon/Wed/Fri/Mon/Wed schedule, using either 7 or 9 Gy per fraction. Tumour growth inhibition was studied by repeat MR images acquired over ≈4 months, post-SBRT. Histological analysis of normal tissues (liver, kidney, small and large intestines) after sacrifice was performed to assess normal tissue damage.

Subsequently, the SGPC35-implanted mice were irradiated using the same schedule with 8 Gy per fraction and imaged pre- and post-SBRT with FAZA-PET to measure reoxygenation. This SBRT regimen is currently being offered to pancreatic cancer patients at our institution as part of a phase III clinical trial^68^. The SGPC35-implanted mice were studied in two cohorts. The first cohort comprising 20 mice (12 irradiated, 8 control; only irradiated mice were imaged for hypoxia) was used to assess reoxygenation and radiation-induced tumour growth inhibition. Once it was established that reoxygenation occurred in this tumour model, a second cohort of 10 mice (6 irradiated mice, 4 control) was added to address whether perfusion DCE-MRI measurements could quantify reoxygenation. Irradiated mice in both cohorts were imaged for hypoxia using FAZA-PET before and after SBRT; in the second cohort, control mice were also imaged using FAZA-PET. Tumour volumes were tracked in the first cohort for up to ≈7 months after the end of irradiation using serial T2-weighted MRI (T2MRI). Mice in the second cohort were sacrificed within days of completion of treatment, once FAZA-PET and DCE-MRI imaging had been completed. For all groups, animals were prepared such that irradiation began once the tumours were between 50 and 200 mm^3^ in volume. Table 1 shows the detailed imaging and irradiation schedule for both cohorts of SGPC35-implanted mice around the time of radiotherapy.

**Table 1:**
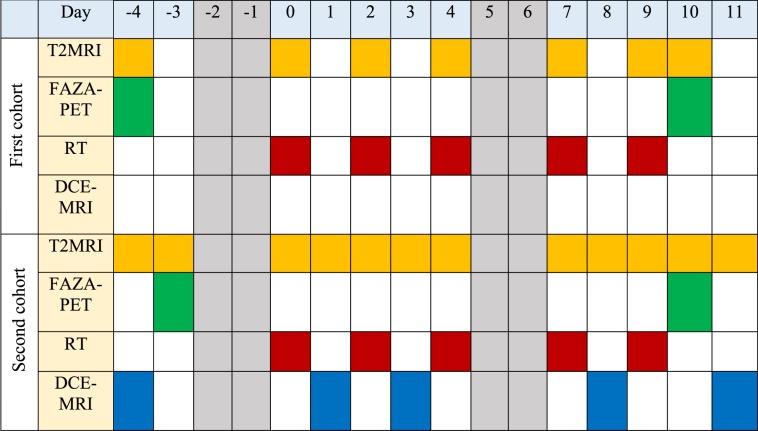
Imaging and irradiation schedule for both cohorts of mice around the respective times of their radiotherapy (RT). Grayed-out regions indicate weekends.

### Magnetic resonance imaging

Magnetic resonance imaging was used to assess tumour volume before and after irradiation, to provide tumour visualization for image-guided irradiation, and also for perfusion quantification using contrast-enhancement. All images were acquired on a Biospec 70/30 USR 7 Tesla preclinical system (Bruker Corporation, Ettlingen, DE) with the B-GA12 gradient coil insert and a 3.5 cm diameter quadrature volume coil of Bruker manufacture. Imaging was performed on mice in prone position on a custom slider bed, with anaesthesia delivery (1.8% isoflurane in oxygen) via nose cone. For DCE-MRI, a pneumatic pillow was fixed anterior to the mouse for respiratory motion suppression and respiratory monitoring, using the SA Instruments system (SAII, Stony Brook, NY). On treatment-only days, the pneumatic pillow was not implemented to improve geometric consistency with planning cone-beam computed tomography (CBCT) images (see Section 2.4) used for treatment.

For all T2MRI scans (image guidance and tumour volume monitoring), tumors were visualized using a multi-slice 2D-T2-weighted RARE technique in the transverse plane (echo time 8 ms; RARE factor 10; effective echo time 40 ms; repetition time 3 sec; 140 × 140 matrix over 28 × 28 mm field-of-view for 200 um in-plane resolution up to 32 contiguous 1 mm slices; 5 averages; 7 min scan time).

DCE-MRI was performed using the contrast agent Gd-DTPA (Gadovist, Bayer Corporation, Leverkusen, DE) at a dose of 15 µl per mouse. DCE-MRI data were then acquired using 3D-FLASH in a transverse plane centered on the tumor (echo time 3 ms; repetition time 8.33 ms; 12° flip angle; 112 × 100 × 12 matrix over 28 × 25 × 12 mm field-of-view for 0.25 × 0.25 × 1 mm^3^ spatial resolution; 10 s temporal resolution; 36 repetitions; 6 min scan time). After 3 repetitions, 15 ul of Gd-DTPA was delivered via bolus injection through the tail vein using an automated MR-compatible syringe pump (PHD 2000, Harvard Apparatus, Holliston, MA).

### Tumour volume measurements

T2MRI image sets were imported into RayStation 6.0 (RaySearch Laboratories, Stockholm, Sweden) and tumours were manually contoured by one of the authors (AEA) in 3D to quantify volumes.

### Irradiation

Mice were irradiated with an X-RAD 225Cx small animal irradiator (Precision X-ray, North Branford, CT) using a 180° arc and 8, 10, or 15 mm diameter collimators, depending on tumour size. The system was calibrated following the TG-61 protocol^69^ at 225 kV peak voltage. For image guidance and sizing of the tumors, T2MR images were acquired before irradiation as determined using the pre-irradiation MRI acquisition. The mice were then transferred to the X-RAD and re-imaged using the integrated CBCT imaging system. The two image sets were registered to assure precise alignment of the treatment fields with the target^70^. Collimators were chosen to give a $\gtrsim$2 mm geometric margin, ensuring coverage of the target in the presence of residual setup uncertainty. One treatment plan was generated for each collimator and prescription dose (7, 8, or 9 Gy per fraction for 5 fractions), with dose-rate/beam-on time specific to the collimator and prescription. Individual treatment plans for each animal were not created; the treatment plan assumed a generic animal size and anatomy. An example dose distribution calculated using SmART-Plan^71^ is shown in Fig. 1a,b.

### FAZA-PET and CT imaging

FAZA was produced by the Centre for Prove Development and Commercialization (Ontario, Canada). Mice were anesthetized with 2% isoflurane in medical air (1.0 L/min) and injected intravenously via the tail vein with 15 MBq of FAZA (0.55 ± 0.07 MBq/g of body weight). PET images were acquired on a Focus 220 microPET scanner (Siemens Preclinical Solutions, Knoxville, TN) at 2 hours post-injection using a Minerve triple-mouse imaging bed (Esternay, France) with controlled heating to maintain body temperature at 37 °C. The PET acquisition consisted of a 20-minute emission scan followed by a transmission scan using a ^57^Co source used later for attenuation correction. Immediately after small-animal PET, the Minerve bed was transferred to an eXplore Locus Ultra preclinical computed tomography (CT) scanner (GE Healthcare, London, ON, Canada) where mice were imaged with routine acquisition parameters of 80 kV and 50 mA to facilitate anatomical delineation for FAZA-PET quantification.

### FAZA-PET hypoxic fraction

The T2MRI, CT, and PET datasets were imported into Inveon Research Workspace (IRW 4.0, Siemens, Knoxville, USA). There was significant anatomical deformation between the T2MRI and PET/CT datasets and only the PET and CT datasets were fused (manually). At the same time, tumours were difficult to visualize on CT images and the tumour volumes were contoured on T2MRI. Tumours were considerably more rigid than surrounding normal tissues and it was assumed that their shape and volume did not change between these scans. The T2MRI-derived tumour contours were thus copied to the PET/CT datasets and positioned using anatomical landmarks visible in both sets of images (kidney, bowel, stomach, duodenum). In cases where the tumour was abutting the bowel and the bowel exhibited significant uptake of FAZA, to correct for spillover effects, tumour contours were eroded by ≈2 mm along the tumour-bowel interface. Left ventricle and spinal muscle regions of interest (ROIs) were also contoured on CT scans for hypoxia quantification.

The imaged FAZA activity for each PET voxel inside tumour ROIs was divided by the mean imaged activity in the left-ventricle, giving the voxel-scale tumour-to-blood ratio (TBR). Hypoxia was quantified by three metrics: mean tumour-to-blood ratio TBR across the entire tumour; the fraction HF_1.4_ of voxels in a tumour for which TBR >1.4^72^; and the fraction HF_3σ_ of voxels for which TBR > 2.4, where 2.4 is three times the standard deviation of the pooled muscle activity data, where the voxel-scale activity data for each mouse muscle set was normalized such that the mean value for each mouse was the same^73^. Reoxygenation was quantified by the changes in hypoxic fraction (${\Delta {\rm{HF}}}_{\Lambda ,{\rm{measured}}}$) between the pre- and post-SBRT FAZA-PET image acquisitions.

To assess the impact of reoxygenation on tumour growth inhibition, irradiated tumours were divided into two groups based on whether ${\Delta {\rm{HF}}}_{\Lambda ,{\rm{measured}}}$ was greater or less than its median value; *p* values for the relative tumour growth at several time points after completion of SBRT in these two groups were calculated using the Mann-Whitney *U* test.

### PET-MRI measurement of reoxygenation

Stratification of individual voxels in a dynamic contrast enhanced image between perfused and unperfused states was achieved by modeling the probability distribution of MRI voxel-scale signal enhancement as a bimodal distribution (Eq. 1). One advantage of this approach is that it may be insensitive to factors affecting MRI signal non-linearity with contrast concentration, and therefore with vascular density.

1 $$P[\Delta S]=\frac{1}{\sqrt{2\pi }}[{v}_{u}{e}^{-{(\Delta S-{S}_{1})}^{2}/2{\sigma }_{1}^{2}}+(1-{v}_{u}){e}^{-{(\Delta S-{S}_{2})}^{2}/2{\sigma }_{2}^{2}}],$$

Here, $\Delta S$ is the measured per-voxel signal enhancement post-contrast injection, ${v}_{u}$ is the volume fraction of unperfused tissue, *S*_1_ and *S*_2_ are the fitted signal enhancements in the unperfused and perfused regions, and σ_1_ and σ_2_ are the Gaussian distribution widths. ${v}_{u}$, *S*_1_, *S*_2_, σ_1_ and σ_2_ were determined for each tumour by fitting Eq. 1 to histograms of voxel-scale enhancement data. Examples of bimodal fits to signal enhancement in orthotopic pancreas tumours are shown in Fig. 5d–f.

We hypothesized that changes in ${v}_{u}$ throughout treatment might then predict for changes in hypoxic fractions measured using PET, as follows:

2 $${{\rm{HF}}}_{\Lambda }={v}_{u}{{\rm{HF}}}_{u}+(1-{v}_{u}){{\rm{HF}}}_{0}.$$

Here, ${{\rm{HF}}}_{\Lambda }$ is the hypoxic fraction for a given PET-hypoxia TBR threshold $\Lambda$, and ${{\rm{HF}}}_{u}$ and ${{\rm{HF}}}_{0}$ are the hypoxic fractions assigned to unperfused and perfused volume fractions measured using MRI.

We then assume that the metabolic rate of oxygen consumption inside each compartment is consistent through treatment, so that a change in ${v}_{u}$ will result in a change ${\Delta {\rm{HF}}}_{\Lambda }$ (Eq. 3) in hypoxic fraction:

3 $${\Delta {\rm{HF}}}_{\Lambda }\approx \Delta {v}_{u}({{\rm{HF}}}_{u}-{{\rm{HF}}}_{0}).$$

In practice, the values of ${{\rm{HF}}}_{u}$ and ${{\rm{HF}}}_{0}$ depend on the choice of $\Lambda$ as well as tumour biology, notably oxygen metabolism, and will vary from tumour to tumour (patient to patient in the clinic). The assumption of constant oxygen metabolism in each compartment is not equivalent to an assumption of constant tumour metabolism, only that changes in metabolism can be accounted for by changes in the occupancies of these compartments. The same assumption has been made in the radiotherapy response modeling of Jeong *et al*.^17^ (see also ref. ^28^), albeit with two hypoxic compartments corresponding to different metabolic sub-levels.

In principle, ${{\rm{HF}}}_{u}$ and ${{\rm{HF}}}_{0}$ for a tumor may then be determined by quantitative analysis of co-registered contrast-enhanced MR and hypoxia-PET image sets. However, accurate regional spatial co-registration is challenging, and a hypoxic fraction in the perfused regions of close to zero can be assumed instead $({{\rm{HF}}}_{0}\approx 0)$. Equation 3 may then be solved for reoxygenation (e.g. ${\Delta {\rm{HF}}}_{\Lambda })$ by dividing HF_*a*_ from the pre-SBRT PET scan with the difference in $\Delta {v}_{u}$ between pre- and post-SBRT MRI scans.

The validity of Eq. 3 and the accuracy of this imaging workflow to predict hypoxic fraction were quantified by calculating the Pearson correlation coefficients between $\Delta {v}_{u}$ and ${\Delta {\rm{HF}}}_{\Lambda ,{\rm{measured}}}$, and between ${\Delta {\rm{HF}}}_{\Lambda ,{\rm{measured}}}$ and ${\Delta {\rm{HF}}}_{\Lambda ,{\rm{predicted}}}$.

### DCE-MRI data modelling

To fit the bimodal distribution in Eq. (1) to MRI signal enhancement data, DCE-MRI datasets were imported into MIPAV imaging software (National Institute of Health Center for Information Technology, Bethesda, USA). Tumours were contoured in MIPAV on DCE-MRI images using T2MR images acquired during the same session as guides to delineation. Up to 6 central slices of the 3D-DCE acquisition (12 mm slice package) were utilized for analysis reflecting tumor diameters of less than 6 mm. The impact of noise in the DCE-MRI images was reduced by fitting Eq. (1) to voxel-scale signal enhancement $\Delta {S}_{i}(t)\equiv {S}_{i}(t)-{\bar{S}}_{i}$ data at 7 time-points: *t = *(30, 40, 50, 60, 70, 80, 90) seconds post-injection, where ${S}_{i}(t)$ denotes the signal in the *i*-th voxel at time *t*. ${\bar{S}}_{i}$ is the mean signal in *i*-th voxel, averaged over three time points before contrast injection. The choice of time (60 ± 30) seconds (s) used to analyze signal enhancement data was chosen to maximize sensitivity to the spatial inhomogeneity in perfusion. 60 s post-injection—effectively a portal-phase acquisition—was long enough to reduce the impact of injection time uncertainty but much shorter than the time required to achieve full diffusive equilibration in the tumour, at which point contrast would be uniformly distributed in the tumour, irrespective of perfusion heterogeneity. We compared the fitting parameters we obtained from data acquired at 60 s with data acquired at 120 s and 300 s post-injection, with little appreciable difference.
